# Transient performance analysis of a novel design of portable magnetic refrigeration system

**DOI:** 10.1063/5.0077701

**Published:** 2022-01-20

**Authors:** Uddip Kashyap, Ashish Kumar, Vishal Sardespande, Sandip K. Saha

**Affiliations:** 1 Department of Mechanical Engineering, Indian Institute of Technology Bombay, Mumbai 400076, India; 2 Department of Energy Science and Engineering, Indian Institute of Technology Delhi, Delhi 110016, India; 3 CTARA, Centre for Technology Alternatives for Rural Areas (CTARA), Indian Institute of Technology Bombay, Mumbai 400076, India

## Abstract

The widely used ice chamber-based cold storage for the transportation and storage of vaccines has several disadvantages, including uncontrolled overall temperature, water accumulation, and frequent ice pack renewal. Therefore, in this work, we numerically studied a novel vaccine storage system by coupling magnetic refrigeration and ice packs developed by conserving the advantages of an ice-based system. A two-dimensional numerical model is developed to analyze the magnetohydrodynamic natural convection in the storage chamber. Gadolinium of 0.08 kg is used to produce a cooling power of 31.514 W and a coefficient of performance of 1.3. With the constant heat leaked of 0.828 W into the system with dimensions of (0.1 × 0.1) m, the average life of the ice pack of 0.75 kg is 1.03 h. By introducing the magnetocaloric effect, the life of the same ice pack can be infinite with no load. The dynamic mode decomposition analysis reveals that the most dominant fluid interaction occurs between the cooled gadolinium plate and the adjacent fluid, resulting in efficient cooling of the air chamber. The developed vaccine chamber design will significantly improve the existing ice pack system with a nominal increase in cost and system weight.

## INTRODUCTION

Most vaccines need an environment of 2–8 °C to sustain and remain effective. The last mile distribution of vaccines requires proper infrastructure for their effective storage and mobilizing the vaccines to the remote corners of a country. One of the most effective solutions used is the mobile ice-based storage chamber. The construction of such chambers is simple. The outer casing is an insulator, and ice packs are introduced inside the chamber to store the vaccines. However, the ice pack-based vaccine storage system has several drawbacks. The addition of ice adds a considerable payload, which needs to be carried along with the vaccine. The operational time of the ice pack depends on the melting rate of the ice influenced by the external temperature. Moreover, precise controlling of the temperature in the range 2–8 °C is challenging. There is no continuous monitoring of the chamber temperature, making the system solely dependent on the operator's experience. Once the ice melts to water, the water should be removed from the chamber at frequent intervals to avoid adversely acting as an internal heat source to the ice packs. However, the positive aspects of the existing ice pack vaccine storage system are as follows: (i) ease of operation, (ii) high portability, (iii) decent weight of approximately 5 kg during operation, and (iv) affordable operating cost.

Several cooling options for vaccine storage are proposed in the published literature. Various cold storage systems are developed with vapor and absorption refrigeration processes.^1^ The efficiency of the vapor refrigeration system is about 40% as compared to the Carnot cycle. Further, the system consists of an absorber, evaporator, condenser, and compressor, making the system bulky. As a result, using vapor refrigeration will, in turn, add weight to the setup. Similarly, the vapor absorption system requires an ammonia–water and hydrogen gas cycle with a heating source and a suitable pump, which increases the complexity of the system.^2^ Moreover, incorporating such processes in a miniature form incurs a high cost. Few recent works use thermoelectric cells to cool the chamber.^1^ Thermoelectric cells work on the principle of Seebeck effect. When current is passed, it induces hot and cold regions on opposite faces. The drawbacks of thermoelectric cells are as follows: (i) They consume a high amount of power. (ii) Their life cycle is limited.^1^ These disadvantages limit the usage of thermoelectric cells in portable vaccine storage systems. Therefore, closed systems with ice packs are still effectively used as a vaccine storage system. However, its efficiency and longevity can be enhanced. Thus, the magnetic field is employed in the present study to improve the performance of the vaccine storage system. One of the recent experimental works by a group of researchers showed that adding an external magnetic field to an aqueous fluid containing magnetic micro-particles results in a chained microstructure.^3^ Further, the interfacial behavior in magnetic multiphase flows was numerically studied by a group of authors.^4^ The lattice Boltzmann method was coupled with a magnetic field to observe such an effect. It was reported that convective flow patterns are enhanced under the influence of magnetic field.^4^ Further, the merging of ferrofluid droplets along with oil was studied.^5^ It showed that the elongation of these droplets along the magnetic fields results in a positive impact on the merging process.^5^ Another work used a magnetic field for a nano-scale fabrication process.^6^ As noted earlier, the addition of magnetic particles induces a chain-like structure. The strength of the magnetic field^6^ determines the average length of such a structure.

The above literature study establishes that the convective flow patterns may be changed using magnetic nanoparticles in the presence of a magnetic field. The length of the chain-like structure depends on the strength of the magnetic field. Thus, magnetic field plays an important role in determining the heat transfer rate. Moreover, a magnetic field produces a heating and cooling effect when a suitable metal is used. This phenomenon is known as the magnetocaloric effect (MCE),^7^ where no movement of nanoparticles is present. The application of a magnetic field reduces the heat capacity of gadolinium, and its de-magnetization again enhances the heat capacity of the material. Thus, heat is released during magnetization, which is absorbed immediately, and the de-magnetization of gadolinium absorbs heat from the surroundings, thereby producing a cooling effect.^7^ In this work, magnetic refrigeration is incorporated in a traditional ice-based chamber. The efficiency of the magnetic refrigeration system is 60% as compared to the Carnot cycle.^7^ Magnetic refrigeration uses the magnetocaloric effect (MCE) to create a lower temperature, which was first observed by Warburg.^8^ The MCE originates from the combination of the magnetic lattice and change in the magnetic field.^9^ The entropy of the magnetic material changes when its electrons are in line with the magnetic field.^10^ This results in a change in the temperature of the magnetic material. Thus, at constant pressure, the entropy of the magnetic material is a function of both the magnetic field and temperature.^10^ The total entropy is a sum of the contribution from magnetic $S_{\mathrm{mag}}$, lattice $S_{\mathrm{lat},}$ and electronic $S_{\mathrm{ele}}.$^10^

Currently, a series of designs based on the MCE is proposed.^10^ A rotary-based setup with 1.2 kg Gd was proposed by Aprea *et al..*^11^ The authors achieved a cooling power of 190 W and a coefficient of performance (COP) of 0.6. Gao *et al.*^12^ integrated pressure-based refrigeration with active magnetic refrigeration. They observed a cooling power of 40 W with a maximum magnetic field of 1.4 T. Another rotary-based system was developed by Huang *et al..*^13^ The applied magnetic field was about 0.875 T over Gd of 1.18 kg. The cooling power of the setup with no load was found to be ∼162.4 W. Further, Aprea *et al.*^14^ used combined magnetic refrigeration using 20 kg of Gd with a geothermal probe.^14^ The authors obtained a cooling capacity of 190 W with a *COP* of 2.20. One of the significant works by Tishin^15^ evaluated the variation of magnetic entropy of the considered materials with respect to the Debye temperature. The results are reported by considering the varying magnetic field strength and compared with experimental observations. The mean-field theory was used to predict the thermal properties. Further, several authors have developed a one-dimensional transient numerical model of magnetic refrigeration.^16^ The authors reported a variation in the adiabatic temperature and specific heat of Gd as a function of the material temperature and magnetic field intensity.

Thus, from the literature survey, it is revealed that extensive work is reported on magnetic refrigeration. However, the magnetic refrigeration technique is still in the developing phase, and its cooling power and *COP* largely depend on the design of the system. The published literature has reported no such work combining ice chamber and magnetic refrigeration. Hence, a novel vaccine storage system is proposed in this study by coupling magnetic refrigeration and ice packs. The hybrid system dynamics containing both ice and magnetic cooling are studied using a finite volume method-based numerical model. Combining magnetic refrigeration and ice packs aims to remove the disadvantages that arise from using only ice packs inside the chamber. Magnetic refrigeration is used to balance out the leaked heat and the heat load inside the chamber through natural convection to maintain a uniform temperature distribution compared to the traditional ice pack systems. A two-dimensional finite volume method-based numerical model is developed and validated with the existing literature. The magnetocaloric effect is imposed through the source term, which is a function of the magnetic and lattice entropy of Gd, in the temperature equation for Gd. The obtained value of magnetic and lattice entropy over a temperature range is validated with the published literature. The resulting numerical model is used to obtain the temperature field in the Gd sample during magnetization and de-magnetization, which is applied to maintain a constant low temperature in the vaccine chamber. Moreover, understanding the fluid interactions that arise due to temperature gradients is essential. Such insights are obtained using the method of dynamic mode decomposition (DMD). Using the findings from this study, a more effective design can be developed, for enhancing the effectiveness of ice-based MCE systems.

## PHYSICAL DOMAIN

**FIG. 1.**
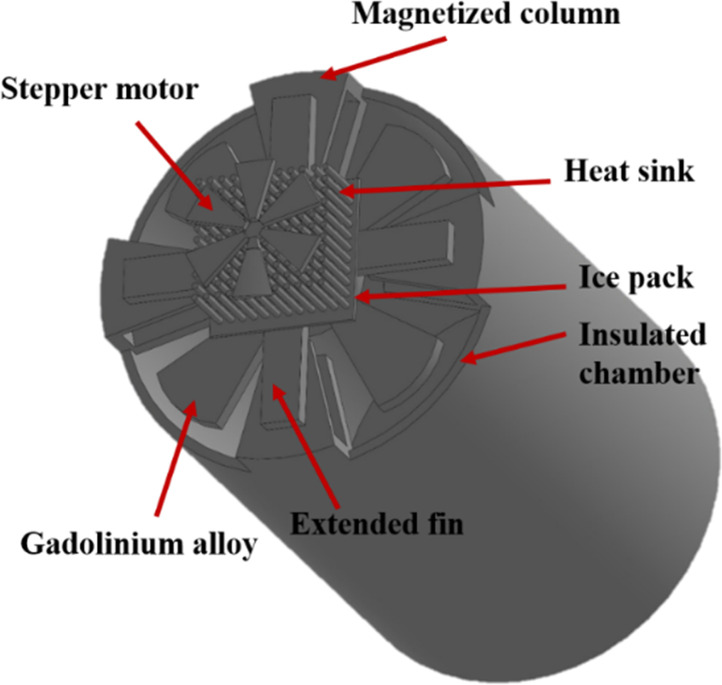
Schematic diagram of the proposed setup.

A detailed design of the proposed system is schematically shown in Fig. 1. The chamber of the proposed design having a volume of 3 L consists of a magnetizing column and gadolinium (Gd) plates rotating in a circular form, as shown in the figure. The Gd plates are rotated using a stepper motor, such that in one step, all the plates of Gd align themselves under the magnetizing column. All the Gd plates are placed out of the magnetizing column in the next step. Thus, the first step is responsible for the magnetization of the Gd plates, and the second step is responsible for their de-magnetization. However, during the magnetization process, the heat capacity of the Gd plates shrinks, resulting in heat loss. This heat is absorbed by the magnetization column and is transferred to the extended fin using conduction heat transfer and finally transported to the vicinity of the ice pack. This constitutes the secondary loop. In the primary loop, after the Gd plates move to the second step for de-magnetization, the heat capacity of the material increases and absorbs heat from the surrounding. Due to this, natural convection inside the chamber starts to occur, where heated air rises to the cooled top of the chamber and cools, and heavier air reaches the heated bottom surface. Thus, a uniform distribution of temperature can be achieved. Initially, the chamber contains air at 2 °C. Heat transfers from the surrounding, which is at a higher temperature, to the chamber through the walls, resulting in a rise of air temperature in the chamber. Due to the effect of natural convection heat transfer, heated air rises from the bottom of the vaccine chamber. Hence, the Gd alloy is placed at the top of the chamber to provide a magnetic cooling effect in the vaccine chamber. The top wall of the vaccine chamber acts as a thermal barrier during the magnetization and de-magnetization processes. The thermal barrier is placed to restrict heat transfer from the Gd to the vaccine chamber during the magnetization process. After the de-magnetization process, the thermal barrier is removed to extract the extra heat generated in the vaccine chamber. This mechanism is achieved with the help of a stepper motor assembly. A single cycle of operation consists of four steps. In the first step, heat is allowed to transfer to the chamber from the surrounding. Once the temperature of the vaccine chamber reaches 7 °C, the magnetization of Gd is carried out in the second step for 0.1 s with heat continuously being lost from the system. The material with thermal conductivity of about 0.12 W m^−1^ K^−1^ placed between Gd and the chamber acts as an insulator.^17^ A secondary loop is activated during magnetization where the generated heat from the surface of Gd is carried away with the help of extended fins to an ice pack placed above using the conduction mode of heat transfer. Then, the de-magnetization of Gd is carried out, which decreases the temperature of Gd, keeping the other boundary conditions similar to the previous step. In the last step, the top wall of the chamber is allowed to exchange heat from Gd to keep the temperature of the vaccine chamber within desired limits.

## MATHEMATICAL MODELING

**FIG. 2.**
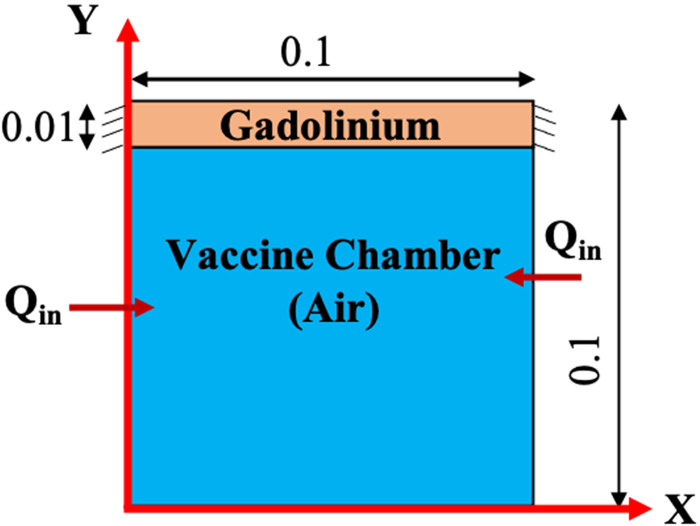
Schematic diagram of the computational domain (all dimensions are in m).

A two-dimensional square cavity is selected to demonstrate the magnetic cooling effect in a vaccine storage chamber with dimensions 0.1 × 0.1 m, as shown in Fig. 2. A mathematical model is developed to investigate the heat transfer characteristics of air in the vaccine chamber during the magnetization and de-magnetization processes of Gd. Few assumptions are made in the process of development of the numerical model as follows: (i) The air present in the vaccine chamber is considered incompressible and Newtonian. (ii) The thermophysical properties of air and Gd over the chosen range of temperature are considered isotropic and constant. (iii) Heat transfer by radiation is neglected. The effect of buoyancy is incorporated in the *z*-momentum equation by using the Boussinesq approximation, which assumes no variation in the thermophysical properties other than the air density. Following are the governing equations that are solved for the fluid (air) and solid (Gd):

(a) Conservation of mass,
$$\frac{\partial \rho_{a}}{\partial t}+\nabla .\left(\rho_{a}V\right)=0.$$ (1)
(b) Conservation of momentum for air,
$$\frac{\partial \left(\rho_{a}V\right)}{\partial t}+\nabla .\left(\rho_{a}V\;V\right)=-\nabla p+\nabla .\left(\nabla V\right)+S_{z}.$$ (2)
(c) Energy equation for air,
$$\frac{\partial \left(\rho_{a}c_{pa}T_{a}\right)}{\partial t}+\nabla .\left(\rho_{a}c_{pa}VT_{a}\right)=\nabla .\left(k_{a}\nabla T_{a}\right).$$ (3)
(d) Energy equation for Gd,
$$\frac{\partial \left(\rho_{gd}c_{p,gd}T_{gd}\right)}{\partial t}=\nabla .\left(k_{gd}\nabla T_{gd}\right)+S_{\mathrm{mce}}.$$ (4)

Here, the subscripts *a* and *gd* denote air in the vaccine chamber and Gd, respectively. The source term in the conservation of momentum equation signifies the Boussinesq approximation in the *z*-direction, which is expressed as follows:

$$S_{z}=\rho g\beta \left(T_{a}-T_{\mathrm{ref}}\right),$$ (5)

where β is the thermal expansion coefficient and $T_{\mathrm{ref}}$ is the reference temperature. A volumetric source term is defined to account for the magnetocaloric effect for Gd. The source term is a function of the temporal variation of magnetic flux intensity and adiabatic change in temperature. The volumetric source term can be written as follows:

$$S_{\mathrm{mce}}=\rho_{gd}c_{p,gd}\frac{\partial \left(\Delta T_{ad}\right)}{\partial B}\frac{\partial B}{\partial t},$$ (6)

where B is the magnetic flux intensity and $\Delta T_{ad}$ is the adiabatic temperature. The adiabatic temperature depends on the total entropy of the magnetic material and the specific heat of the magnetocaloric material and can be expressed as follows:

$$\Delta T_{ad}=\frac{T_{gd}}{C\left(B,T_{gd}\right)}S_{\mathrm{total}}(B,T_{gd}),$$ (7)

where $C\left(B,T_{gd}\right)$ is the specific heat of Gd under the influence of magnetic flux as expressed in Eq. (7) and $S_{\mathrm{total}}$ is the total entropy of the magnetic material. $C\left(B,T_{gd}\right)$ is defined as follows:

$$C\left(B,T_{gd}\right)=T{\left(\frac{\partial S_{\mathrm{total}}}{\partial T}\right)}_{B}.$$ (8)

The total entropy of the magnetic material is a sum of the magnetic entropy ( $S_{\mathrm{mag}})$, lattice entropy ( $S_{\mathrm{lat}}$), and electron entropy ( $S_{\mathrm{ele}}$). The effect of electron entropy is marginal as compared to the other two entropies.^15^ Hence, electron entropy is not considered in the present study. The magnetic entropy can be given by

$$S_{\mathrm{mag}}=R\left[\ln\left(\sinh\left(\frac{2J+1}{2J}X\right)\right)-\ln\left(\sinh\left(\frac{X}{2J}\right)\right)-XB_{j}\left(X\right)\right],$$ (9)

where J is the total angular momentum of the magnetic material and $B_{j}\left(X\right)$ is the Brillouin function, which can be expressed as follows:

$$B_{j}\left(X\right)=\frac{2J+1}{2J}\cot h\left(\frac{2J+1}{2J}X\right)-\frac{1}{2J}\cot h\left(\frac{X}{2J}\right).$$ (10)

The value of X can be estimated as follows:

$$X=\frac{G\mu_{b}JB}{K_{b}T_{gd}}+\frac{3T_{cu}JB_{j}\left(X\right)}{T_{gd}(J+1)},$$ (11)

where *G*, $\mu_{b}$, $K_{b}$ and $T_{cu}$ represent the Lande factor, Bohr magneton, Boltzmann constant, and Curie temperature, respectively. Equations (10) and (11) can be solved iteratively in order to obtain the Brillouin function $B_{j}\left(X\right)$ and, subsequently, the magnetic entropy can be estimated. The lattice entropy can be defined as follows:

$$S_{\mathrm{lat}}=R\left[-3\ln\left(1-e^{\frac{T_{de}}{T}}\right)+12\left(\frac{T}{T_{de}}\right)\int_{0}^{\frac{T_{de}}{T}}\frac{x^{3}}{e^{x}-1}dx\right],$$ (12)

where $T_{de}$ is the Debye temperature, which is taken as 173 K.^15^ A numerical integration technique is adopted to estimate the integral term of lattice entropy.

An evaluation of the amount of heat leaking into the system through the insulated wall is important. The heat leak $\left(Q_{l}\right)$ into the vaccine chamber is assumed as 0.828 W. The cooling power $\left(Q_{c}\right)$ and heat rejected $\left(Q_{r}\right)$ from Gd during magnetization are calculated using the following relations:^18^

$$Q_{c}=m_{a}c_{a}\left(T_{c1}-T_{c2}\right),$$ (13)

$$Q_{r}=m_{i}c_{i}\left(T_{i1}-T_{i2}\right).$$ (14)

The electric power $\left(Q_{e}\right)$ required for operating the stepper motor can be evaluated using the following relation:

$$Q_{e}=\mathrm{voltage}\times \mathrm{current}.$$ (15)

The *COP* of the current system can be evaluated using the following relation:^10^

$$\mathrm{COP}=\frac{Q_{c}}{Q_{r}+Q_{l}+Q_{e}}.$$ (16)

### Initial and boundary conditions

The initial temperature of the vaccine chamber and the Gd sample is taken as 2 °C. The initial condition is expressed as follows:

T(x,y,0)= 2 °C. (17)

Heat is allowed to be lost from the sidewall of the vaccine chamber from the surrounding. The ambient temperature is chosen to be 27 °C. The bottom wall of the chamber is considered insulated, while at the top wall, Gd is placed. The walls of the Gd sample are insulated, except the top wall, where a constant temperature of 2 °C is applied during the magnetization process.

### Dynamic mode decomposition

In a dynamic system where multiple phenomena co-occur, it is essential to understand and evaluate the dominating phenomenon. This is because, such a phenomenon significantly influences the overall characteristics of the dynamic system. To assess such dominating phenomena, the method of dynamic mode decomposition (DMD) is opted,^19^ as the dynamics of the system changes both spatially and temporally. The DMD algorithm performs a discrete Fourier transform and orthogonal decomposition over the spatially and temporally varying data set, as follows:

$$\frac{dx}{dt}=f\left(x,t;\alpha \right),$$ (18)

where *f*(.), *x(t)*, and α denote the dynamic equation, time-dependent vector, and parameters of the dynamic system, respectively. At a discrete time step of Δt, the properties of the dynamic system are studied. Thus,

$$x_{k\;}=x\left(k\Delta t\right).$$ (19)

Therefore, the properties of the dynamic system for the next interval can be evaluated as follows:

$$x_{k+1\;}=M\left(x_{k}\right),$$ (20)

where *M* corresponds to mapped time flow and k=1, 2,…,m is the time sampled data till the time $t_{k}$. Thus, the vorticity is calculated as follows:

$$y_{k\;}=g\left(x_{k}\right).$$ (21)

The non-linearity in Eq. (18) makes it difficult to solve. As a result, the DMD model is used to evaluate time and spatial variations by approximating the dynamics of the given system. The linearly approximated DMD dynamics $f\left(x,t;\alpha \right)$ is expressed as follows:

$$\frac{dx}{dt}=Ax.$$ (22)

At condition $x\left(0\right)$, it is defined as follows:

$$x\left(t\right)=\sum_{k=1}^{n}\phi_{k}\;\text{exp}\;\left(\omega_{k}t\right)b_{k}=\Phi \;\text{exp}\;\left(\Omega t\right)b,$$ (23)

where $\phi_{k}$ denotes the kth eigenvector, $\omega_{k}$ indicates its eigenvalue, and $b_{k}$ represents the corresponding coefficient of the matrix *A*.

The discrete time sampled data of the approximated equation [Eq. (23)] can be obtained using the sampled data as shown in Eq. (21). Here, a and $x_{1}$ are the matrix of the dynamic system and initial condition, respectively,

$$x_{k+1}=A\left(x_{k}\right),$$ (24)

A=exp(aΔt). (25)

The obtained solution can be expressed as follows:

$$x_{k}=\sum_{j=1}^{nr}\emptyset_{j}\lambda_{j}^{k}b_{j}=\Phi \Lambda^{k}b.$$ (26)

The coefficient of the matrix *b* can be obtained as follows:

$$x_{1}=\Phi b.$$ (27)

Low-rank eigen decomposition of matrix *A* is achieved through DMD. Thus, the least squares fit of the data set, $x_{k},$ is achieved as follows for *m* recordings:

$${\left\|x_{k+1}-A\left(x_{k}\right)\right\|}_{2}.$$ (28)

A correct approximation can only be obtained when the dynamics of the system are decomposed into numerous time scales. The approximated error is minimized for *m* recordings by positioning the two large data matrices using the recorded state. Thus,

$$X=\left[\begin{array}{ccc} | & | & \vdots \\ x_{1} & x_{2} & \cdots \\ | & | & \vdots \end{array}\;\begin{array}{c} |\\ x_{m-1}\\ | \end{array}\right],$$ (29)

$$X^{\prime }=\left[\begin{array}{ccc} | & | & \vdots \\ x_{2} & x_{3} & \cdots \\ | & | & \vdots \end{array}\;\begin{array}{c} |\\ x_{m}\\ | \end{array}\right].$$ (30)

Equation (29) represents the sampled state of the non-linear system. The above matrix can be approximated as follows:

$$X^{\prime }\approx AX.$$ (31)

Using Eq. (31) yields

$$A=X^{\prime }X^{+},$$ (32)

where ^+^ denotes the Moore–Penrose pseudoinverse. This error can be minimized as follows:

$${\left\|X^{\prime }-AX\right\|}_{F},$$ (33)

where ${\left\|.\right\|}_{F}$ denotes the Frobenius norm, i.e.,

$${\left\|X\right\|}_{F}=\sqrt{\sum_{j=1}^{n}\sum_{k=1}^{m}{X_{jk}}^{2}}.$$ (34)

The high-dimensional matrix *A* is reduced to a low-dimensional matrix $\tilde{A}$. The low-dimensional operator $\tilde{A}$ is used in the DMD algorithm to rebuild the leading nonzero eigenvalues. A detailed algorithm is presented in an earlier work.^20^

### Solution procedure

The governing equations are iteratively solved using a finite volume method (FVM)-based commercial software, Ansys Fluent V16.2. The LES model is chosen for this study as this model creates a velocity tensor of both resolved and unresolved scales from the convective terms. Thus, more than one velocity scale can be observed. A first-order upwind scheme is used to compute the magnetic specific heat, and, subsequently, the adiabatic temperature change and volumetric source term are obtained using Eqs. (7) and (6). To incorporate the magnetocaloric effect in Gd, a user-defined function (UDF) is used to calculate the values of different properties. The convergence criteria for the momentum and energy equation are set to 10^−3^ and 10^−6^, respectively.

### Grid independence study and validation

**FIG. 3.**
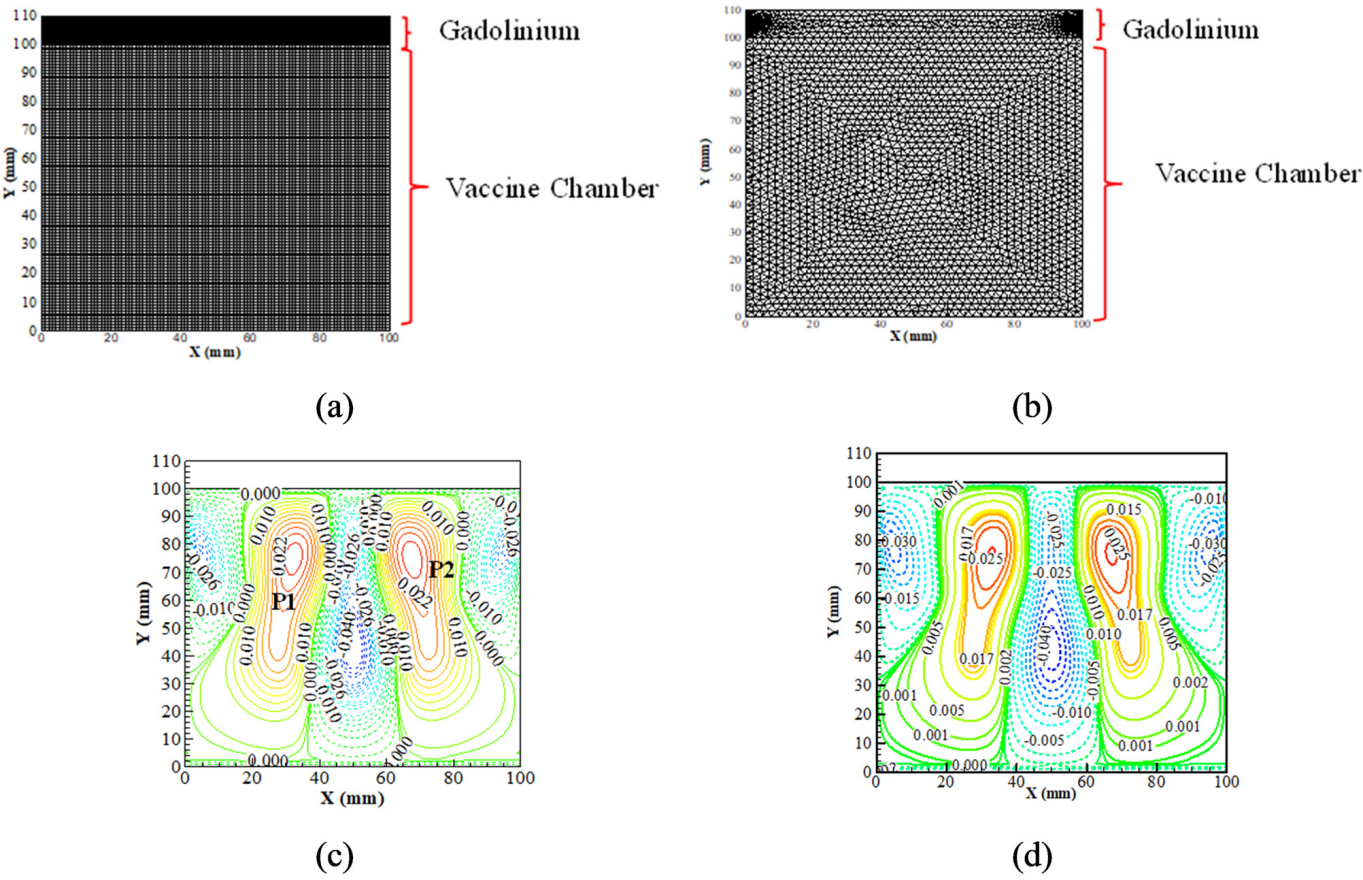
Mesh configuration for (a) quadrilateral and (b) triangular meshes and *v* velocity contours (m/s) for (c) quadrilateral and (d) triangular meshes.

A grid independence study and the current numerical solver validation are carried out prior to performing an extensive study. In order to perform the grid independence test on the two-dimensional rectangular domain, three layouts of the quadrilateral mesh are considered, and the temperature span is compared. The temperature span is considered as the temperature difference between successive time steps. The temperature span at a magnetic field intensity of 1.3 T is evaluated for each grid configuration, as shown in Table I. The variation in temperature with the number of control volumes is minimal. It is found that 2424 control volumes yield a difference in the temperature span of 0.02 °C compared to 3240 control volumes. Therefore, the number of control volumes of 2424 is used for further study. Moreover, as reported in the literature, many LES models exhibit excessive dissipation.^22,23^ Hence, an unstructured grid configuration results in a lower rate of error in comparison to a structured grid when a double pair vortex is studied.^24^ We have compared the results obtained from the quadrilateral [Fig. 3(a)] and arbitrary triangular [Fig. 3(b)] meshes that are shown in Fig. 3. In both cases, the almost identical number of cells obtained from the grid independence study is used (Table I). A comparative analysis shows that, for the considered two-dimensional rectangular domain, the variation between the quadrilateral and arbitrary triangular meshes is slight as both cases yield temperature span values of 9.71 and 9.73 °C, respectively. Moreover, the *v* velocity contours (m/s) at the end of the cooling phase (first cycle) are compared for quadrilateral [Fig. 3(c)] and arbitrary triangular [Fig. 3(d)] meshes. The *v* velocity contours register similar interaction profiles.

**TABLE I.** Temperature span with the number of control volumes.

Type of mesh	Number of control volumes	Temperature span (°C)
Quadrilateral	1646	9.65
Quadrilateral	2424	9.71
Arbitrary triangular	2543	9.73
Quadrilateral	3248	9.73

**FIG. 4.**
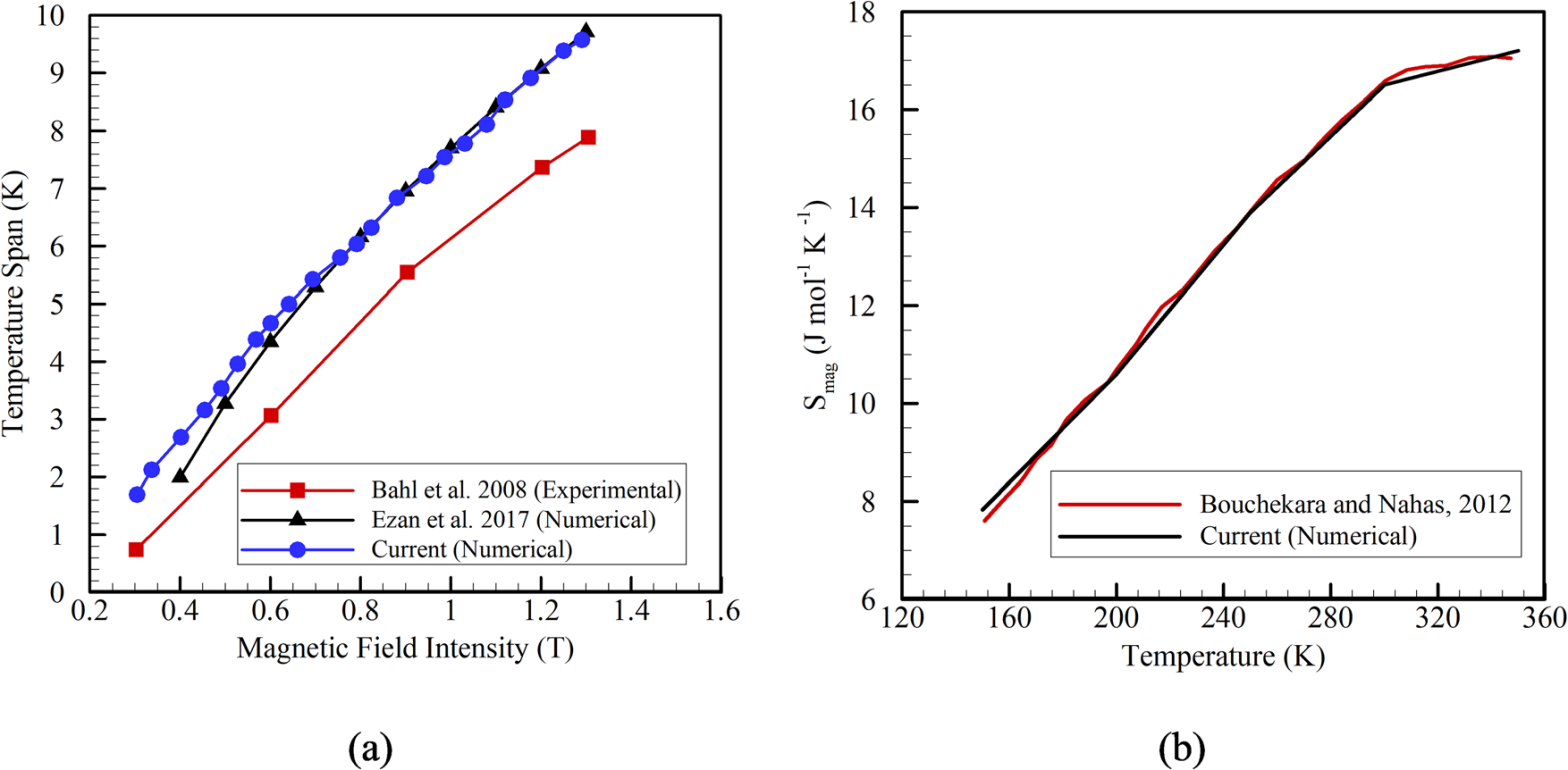
Variation of (a) temperature span with respect to change in magnetic field intensity and (b) entropy $S_{\mathrm{mag}}$ with respect to change in temperature.

After determining the appropriate number of control volumes in the numerical domain, a validation of the current numerical domain is performed. In order to validate, the results obtained from the current numerical analysis are compared with the experimental work of Bahl *et al.*^21^ and the numerical work of Ezan *et al..*^25^ Here, the Gd sample is magnetized from 0.3 to 1.3 T for a duration of 0.1 s. Figure 4(a) reports the variation in temperature span with the magnetic field intensity. When the current numerical results are compared with the experimental results, a constant deviation of 13.7% is observed, though a similar trend is followed. However, identical temperature span profiles are reported when the current numerical results are compared with Ezan *et al.*^25^ The discrepancy between the experimental and the numerical results can be attributed to heat loss from the system during experiments. Thus, the current numerical model can reasonably predict changes in the temperature with the variation in magnetic field intensity.

Further, the current numerical predictions of entropy ( $S_{\mathrm{mag}}$) of the system are compared with the work of Bouchekara and Nahas.^26^ Figure 4(b) reports the variation of $S_{\mathrm{mag}}$ with temperature. The present numerical and published results yield the same values of $S_{\mathrm{mag}}$ with temperature change. Thus, it can be concluded that the current numerical model effectively evaluates and considers the critical physics that influences the physical system. Therefore, the same model and the algorithm are used to analyze the heat transfer performance of the vaccine chamber further.

## RESULTS AND DISCUSSION

**FIG. 5.**
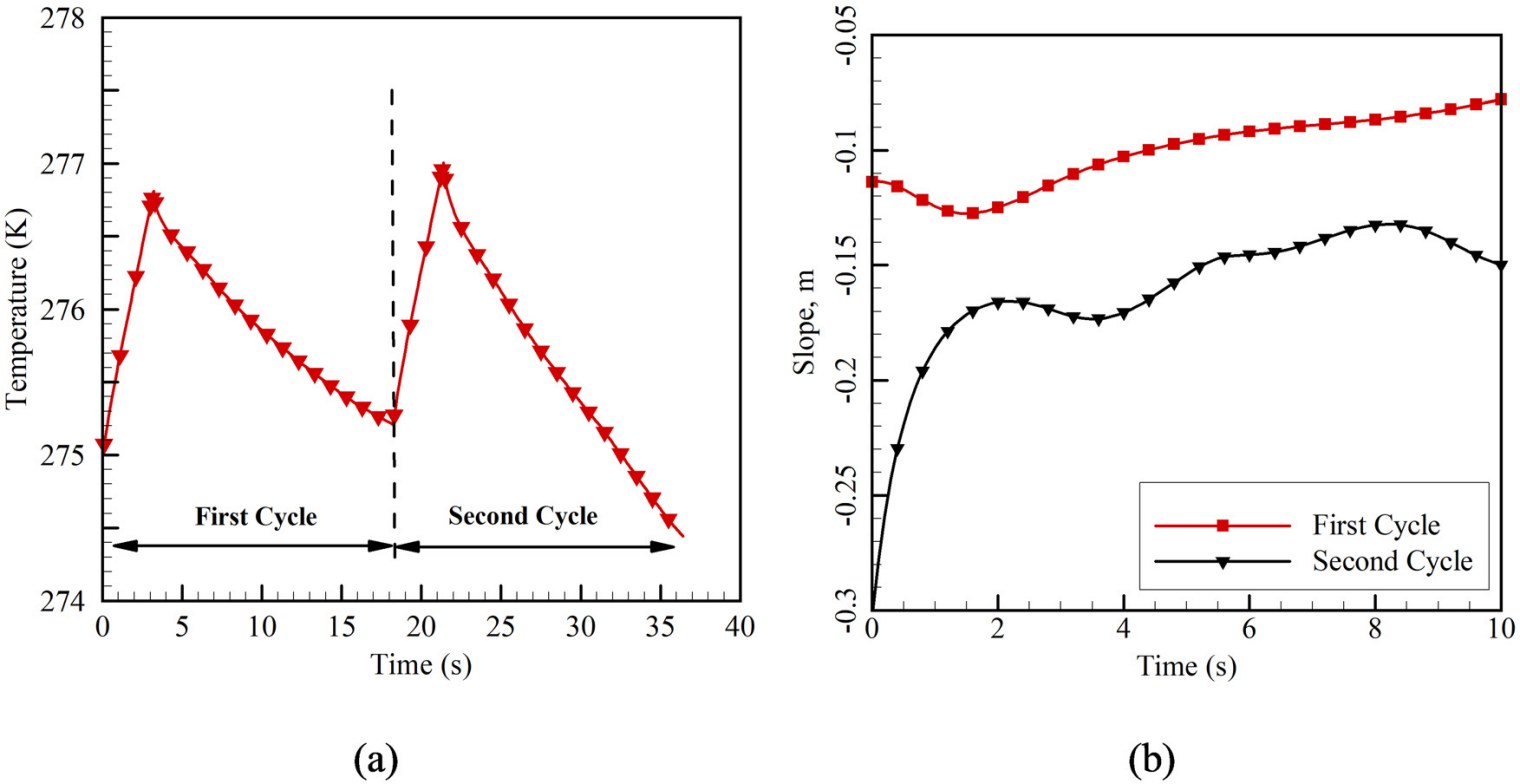
(a) Average temperature of the air domain during the first and the second cycles and (b) comparison of the slope during the cooling period with time for the first and the second cycles.

The study of a hybrid vaccine storage system is performed after validating the numerical model. Both ice-based and magnetic refrigeration-based techniques are used simultaneously to achieve the desired effect of cooling. A finite amount of heat leaks into the vaccine chamber. The overall thermal conductivity of the heat resistant material used in the vaccine storage is 0.12 W/m K.^16^ A heat load of $Q_{l}=$ 0.828 W enters through the three sides of the vaccine chamber, which raises the air temperature in the system by 0.55 K/s. Figure 5(a) shows the change in the average temperature span of the air domain with time for the first and second cycles. The system is initially cooled to 275 K, and heat leakage into the system is allowed. It is observed that within the initial 3 s, the temperature of the chamber reaches about 276.5 K [Fig. 5(a)]. Immediately, a feed is given to the stepper motor, and the Gd blades align themselves inside the magnetization column. The magnetization flux varies from 0.3 to 1.3 T within 0.1 s. The temperature of the Gd sample increases abruptly during this phase.^17^ It is because the electrons in the metallic blades align themselves along the magnetic field. As a result, the heat capacity of the blades decreases, which causes the blades to heat up. Since the process is instantaneous, no thermal response is observed in the surrounding air.^11^ Moreover, the generated heat is transferred to the ice pack through the extended fins by conduction heat transfer.

The blades are moved out of the magnetization column at the next instant, and the magnetic flux varies from 1.3 to 0.3 T in 0.1 s. During the de-magnetization phase, the randomness in the alignment of electrons is restored, and the heat capacity of the metallic blades is increased. As a result, the material absorbs heat from the surroundings, as shown in Fig. 5(a) after 5 s. The blades are allowed to expose to the surrounding air for 13.2 s, which is at 276.8 K at that instant. As a result, after 13.2 s of exposure, the average temperature of the surrounding air reduces to 275 K. The same cycle is again repeated. At the end of the second cycle at 36.4 s, the average temperature of the air chamber reduces to 274.4 K.

Further, the slope of the cooling phase with time is compared for both the first and second cycles. For the first and second cycles, the cooling phases start from 5 and 23.2 s, respectively. The initial 10 s from the start of the cooling phase is considered, and the slope of temperature span change with time is compared, as shown in Fig. 5(b). It is observed that the overall slope of the second cycle is higher than that of the first cycle. During the magnetization phase, heat is generated on the surface of the Gd blades. The ice pack readily absorbs a part of this heat with the help of extended fins. As a result, a lower temperature value is registered at the end of the second cycle, as shown in Fig. 5(a).

**FIG. 6.**
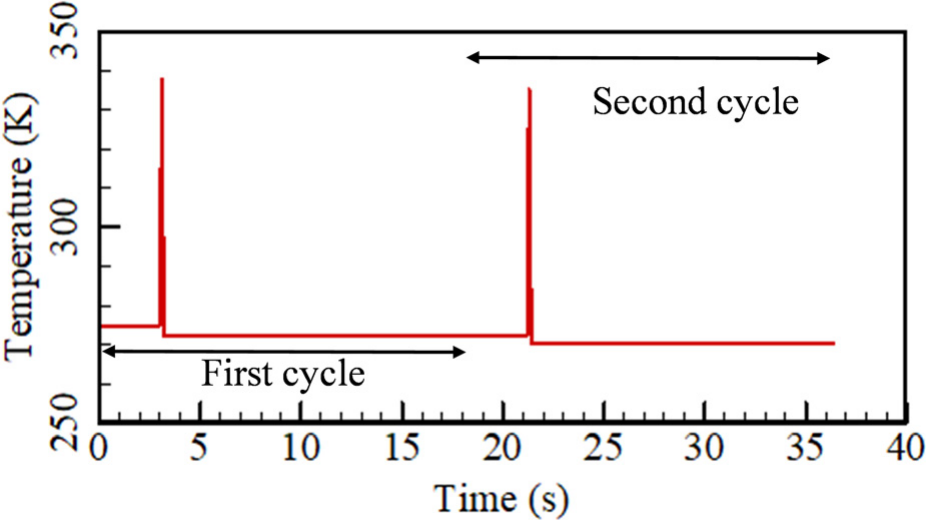
Average temperature of the Gd blades during the first and the second cycles.

With the observation of the average temperature of the air domain, the average temperature of the Gd blades with time is plotted in Figure 6 for the first and second cycles. As magnetization and de-magnetization are instantaneous processes, the variation in temperature of the blades is also dynamic. It is found that a peak in temperature is observed in both first and the second cycles for the magnetization and de-magnetization processes (Fig. 6). For the first cycle, the average temperature of the Gd blades is 275 K. During the magnetization phase, the average temperature of the Gd blades reaches about 337.6 K, and after de-magnetization, the blades achieve an average temperature of 272.5 K (Fig. 6). Due to the change in heat capacity of the Gd blades during the magnetization and de-magnetization processes, an instantaneous variation in temperature of the Gd plates takes place. This temperature variation is due to the transfer of heat to the ice pack during magnetization. Further, the peak average temperature of 335 K is observed during the second cycle. Moreover, at the end of the de-magnetization phase, the Gd blades reach a temperature of about 270.25 K. Thus, the average temperature during both magnetization and de-magnetization phases decreases in the second cycle compared to the first cycle. As a result, at the end of the second phase, the cooling effect initiates due to a temperature difference of about 4.75 K (Fig. 6).

**FIG. 7.**
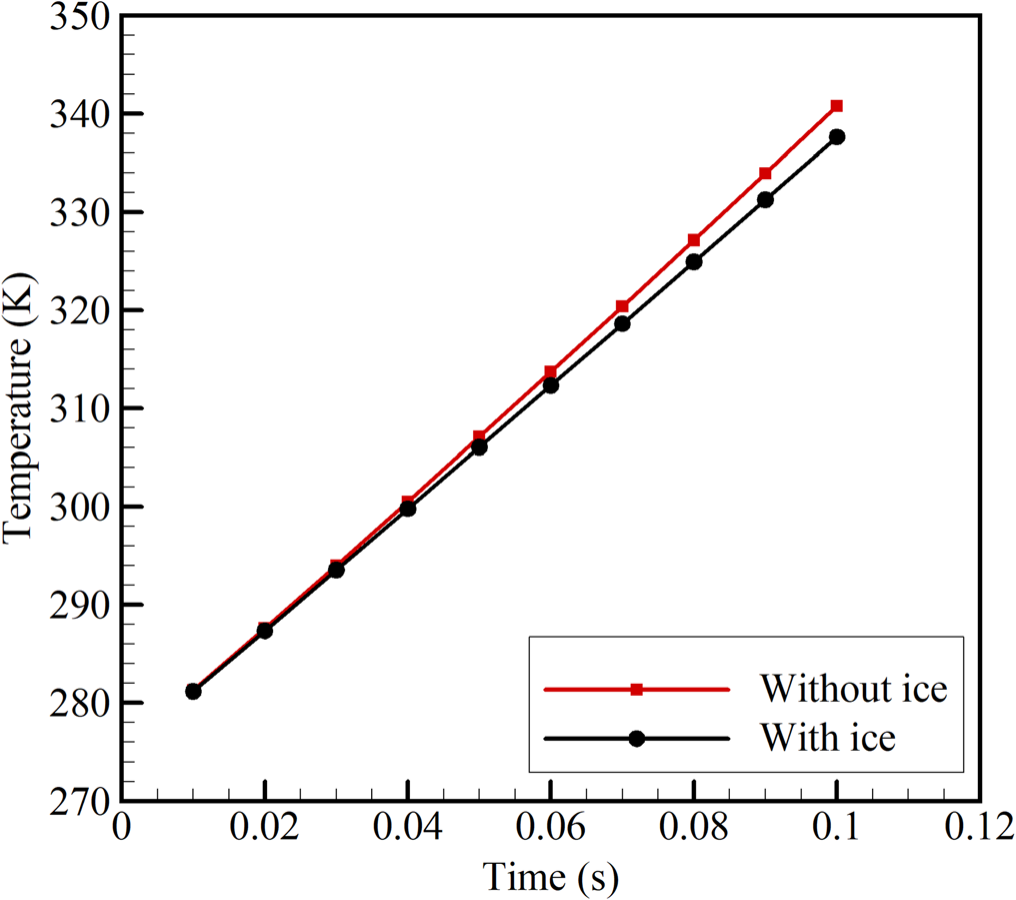
Average temperature of the Gd plates with and without ice packs.

In order to quantify the role of the secondary loop constituting the extended fin and the ice pack, two cases are simulated. The first case consists of no ice pack and the second case consists of 0.75 kg of ice. The magnetization process is instantaneous, and the considered time period is 0.1 s. The magnetic field intensity changes from 0.3 to 1.3 T. Figure 7 shows the transient variation of the average temperature of Gd plates with and without ice packs. Initially, during the start of magnetization (0.01 s), the registered temperature of the Gd plates is 281 K. At the end of the magnetization phase, a substantial temperature change is seen. The case with and without ice pack reports 337.6 and 340.7 K, respectively. The case with an ice pack registers 3.31 K lower than that without an ice pack, although both the cases start from the same initial temperature. This is because heat is transferred to the ice pack during the magnetization phase through the extended fins attached to the magnetization column.

**FIG. 8.**
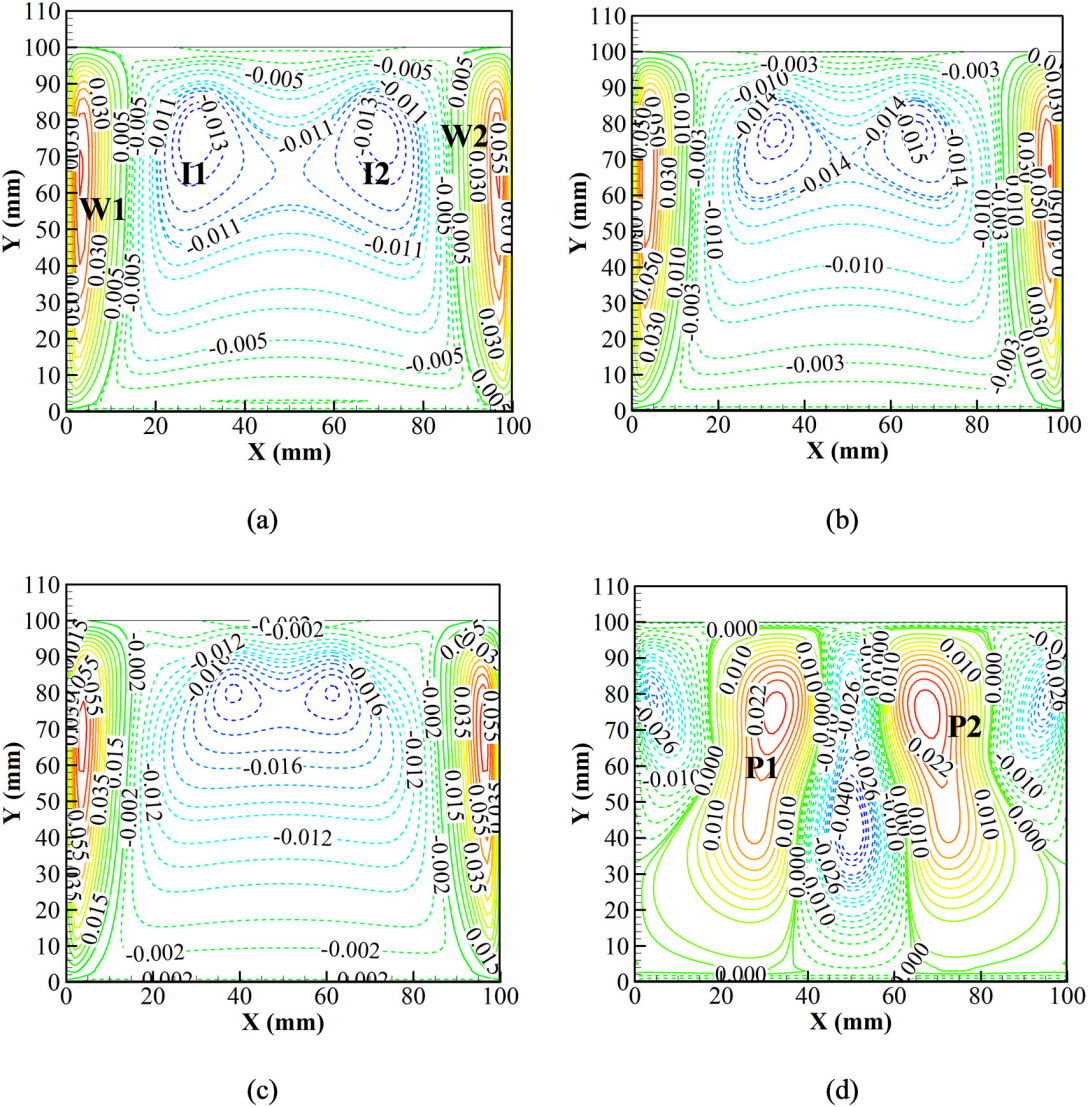
*v* velocity contours (m/s) at (a) initial, (b) magnetization (0.1 s), (c) de-magnetization (0.1 s), and (d) cooling phases of the complete system during the first cycle.

With the observation of average temperature, the *v* velocity and temperature contours of the entire system during each step of the first cycle are evaluated. Initially, the system is kept at 2 °C, and heat is allowed to leak into the system. Figure 8 presents the *v* velocity contour in the vaccine chamber. Figure 8(a) shows a couple of convection cells near the walls (W1 and W2) induced due to heat leakage into the system at 3 s through the side walls. The convection cells, W1 and W2, rotate in the clockwise and anti-clockwise directions, respectively. The wall convection cells are of significant strength, which affects the entire fluid domain. A couple of induced convection rolls, I1 and I2, are formed due to the interactions of convection cells, W1 and W2, respectively. The convection cells, I1 and I2, rotate anti-clockwise and clockwise, respectively. The magnetization (0.1 s) and de-magnetization (0.1 s) processes do not instantaneously change the flow behavior [Figs. 8(b) and 8(c)]. However, the natural convection intensifies during the cooling phase (15 s) [Fig. 8(d)]. Therefore, a couple of convection rolls, P1 (clockwise) and P2 (anti-clockwise), are produced that pull up the heated air, and the cold bulk fluid is pushed down through the central region.

**FIG. 9.**
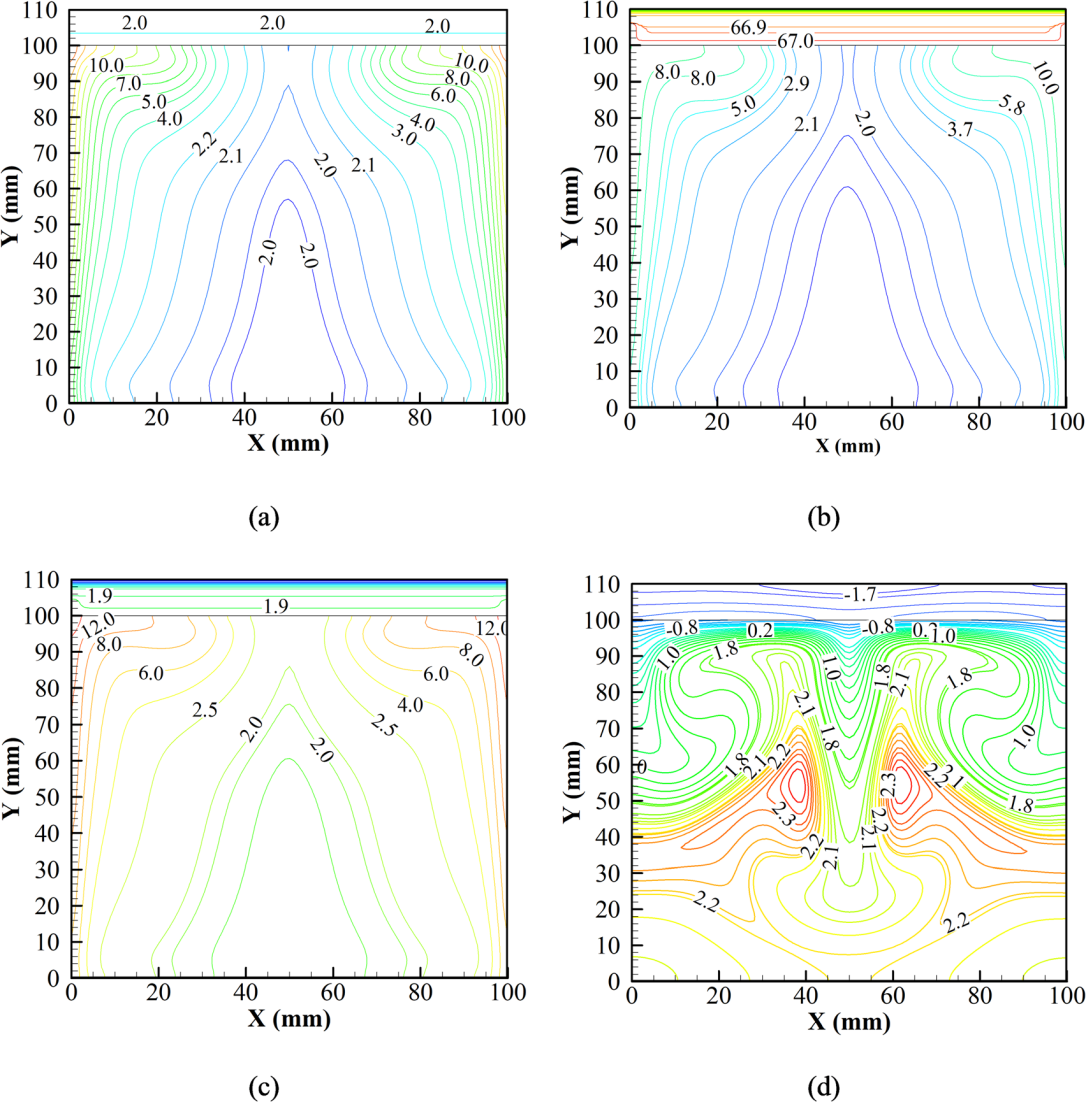
Temperature contours (°C) at (a) initial, (b) magnetization (0.1 s), (c) de-magnetization (0.1 s), and (d) cooling phases of the complete system during the first cycle.

Figure 9 depicts the temperature contours in the vaccine chamber. From Fig. 9(a), it is observed that during the initial 3 s, when heat is allowed to leak into the system, the core air temperature insignificantly changes. The core, as well as the Gd blades, remains at 2 °C initially. However, few isotherms are seen as high as 10 °C near the wall at which heat load is assigned. For the following 0.1 s, magnetization of the Gd blades is initiated by applying a magnetic field intensity of 0.3–1.3 T. An abrupt rise in the temperature of Gd of about 67 °C is observed, as shown in Fig. 9(b). Since magnetization is instantaneous, no temperature rise is observed in the air at the very instant of magnetization. Immediately after the magnetization phase, the de-magnetization phase is initiated for a period of 0.1 s when the magnetic field intensity changes from 0.3 to 1.3 T.

At the end of the de-magnetization phase, an abrupt decrement in the temperature of the Gd plate is reported, as shown in Fig. 9(c). The average temperature of the Gd plate is about 1.9 °C. However, during the end of the de-magnetization phase, the volume of air adjacent to the heat leakage source reaches a temperature of about 12 °C. For the following 15 s, the system is allowed to cool down with the help of natural convection. At the end of the cooling phase, the average temperature of the Gd plate is reported as 1.7 °C [Fig. 9(d)]. A stronger convection cell can be seen in the air domain [Fig. 9(d)]. The previously heated zone of 12 °C is cooled down to about 1 °C. The heated air rises along the side of the chamber, and the cooled air descends at the center of the enclosure. The air witnesses a maximum temperature of about 2.3 °C at the core of the induced convection cells at the central location formed due to natural convection.

**FIG. 10.**
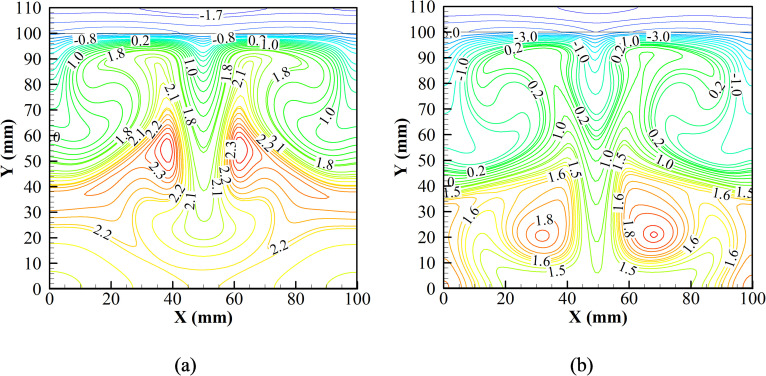
Temperature contours at the end of (a) first and (b) second cycles.

With a thorough evaluation of the temperature behavior at different stages of the first cycle, the temperature contours at the end of the first and the second cycles are evaluated. Figure 10 shows the temperature contours at 18.2 and 36.4 s. As mentioned previously, the heat produced during the magnetization phase is absorbed by the ice pack, which results in a higher cooling slope in the second cycle, as explained above [Fig. 5(b)]. The core of the induced vortices for the second cycle also registers a lower temperature of about 1.8 °C in comparison to 2.3 °C for the first cycle. Moreover, the temperature in the air domain during the second cycle registers a lower value than that during the first cycle, as shown in Fig. 10(b). Further, comparing the temperature profile at the end of the second cycle to that of the first cycle reveals that a lower air temperature is achieved at the end of the second cycle.

With an understanding of the flow and thermal behavior of the system, an effort is made to study the dominance of a particular phenomenon. Since the system is dynamic both spatially and temporally, a DMD analysis is performed to evaluate the dominating dynamics of the system. Evaluating such dynamics will help to carefully design the system and enhance control over the developed system in contrast to the highly complex multi-dimensional phenomena. A further detailed investigation is performed to understand the cause and the dominance of a particular phenomenon in achieving natural convection inside the chamber. The DMD algorithm considers oscillation data of seven periods for an instantaneous velocity field. Prior to the actual evaluation, a study on several data sets and time periods required to predict the dominant modes accurately is to be carried out (Table II). The DMD analysis is executed over the two-dimensional empty space below the Gd plate. The considered sampling times (*t_s_*) are 1, 0.01, and 0.005 s. In this study, a total of 100 data sets is considered. The drawn-out values of the eigenvalues and L_2_ norm are shown in Table II. A maximum deviation of less than 1% is observed for the values of L_2_ norm. Thus, the considered sampling time is chosen as 0.01 s (interaction 1) for further analysis. An insight into the number of data sets is significant. Hence, a data set of *n *=* *50, 100, and 200 is considered (Table III). As shown in interaction 1 (Table III), the maximum deviation in the L_2_ norm is less than 1%. Therefore, the *n *=* *100 data set with a sampling time of 0.01 s is considered for the actual study.

**TABLE II.** Sampling time independence study.

	Interaction 1			Interaction 2			Interaction 3		
*t_s_*	$\lambda_{r}$	$\lambda_{i}$	L_2_ norm	$\lambda_{r}$	$\lambda_{i}$	L_2_ norm	$\lambda_{r}$	$\lambda_{i}$	L_2_ norm
0.05	1	0	3.73 × 10^–6^	0.999	0.009	1.86 × 10^–7^	0.76	0.31	8.47 × 10^–7^
0.1	1	0	3.73 × 10^–6^	0.997	0.011	1.86 × 10^–7^	0.97	0.21	8.47 × 10^–7^
1	1	0	3.69 × 10^–6^	0.869	0.212	1.86 × 10^–7^	0.99	0.04	8.47 × 10^–7^

**TABLE III.** Snapshot number independence test.

	Interaction 1			Interaction 2			Interaction 3		
*n*	$\lambda_{r}$	$\lambda_{i}$	L_2_ norm	$\lambda_{r}$	$\lambda_{i}$	L_2_ norm	$\lambda_{r}$	$\lambda_{i}$	L_2_ norm
50	1	0	3.71 × 10^–6^	0.997	0.016	1.88 × 10^–7^	0.981	0.215	8.44 × 10^–7^
100	1	0	3.73 × 10^–6^	0.997	0.016	1.86 × 10^–7^	0.981	0.215	8.47 × 10^–7^
200	1	0	3.73 × 10^–6^	0.997	0.016	1.86 × 10^–7^	0.981	0.215	8.47 × 10^–7^

**FIG. 11.**
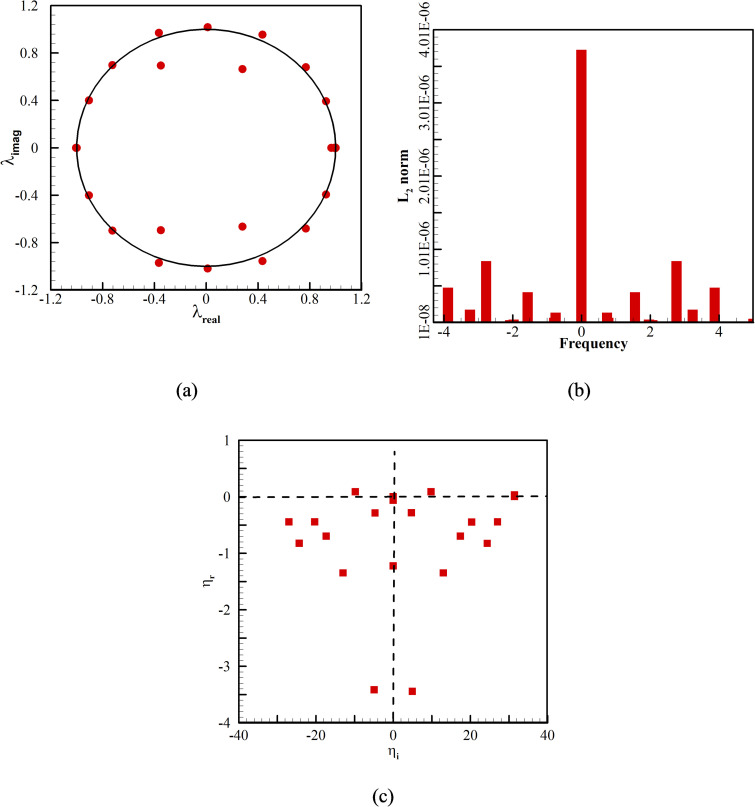
(a) Ritz spectra, (b) energies of the extracted modes as a function of frequency, and (c) the DMD spectrum.

The frequency ( $\eta_{i}$) and the growth or decay rate ( $\eta_{r}$) are evaluated during the cooling phase of the first cycle. To quantify the damped and undamped growth or decay of the induced vortices due to temperature differences, Ritz values are examined.^27^ The values inside the circle define damped vortices, whereas those outside the circle denote undamped vortices. The physical description of such phenomena is as follows. Since DMD is a linear transformation, it defines the magnification or contraction across an eigenvector. The Ritz values during the cooling phase of the first cycle are shown in Fig. 11(a). As can be seen in the figure, the majority or almost all values lie in the circle $\left\|\lambda_{k}=1\right\|$. It shows that the new equilibrium shows negligible modification in the formation of vortices after the transformation. The number of modes present in the system is depicted by bars in Fig. 11(b). The most dominant mode for the system is depicted to occur at $\eta_{i}=0$. The first three modes of the system contribute to most of the energy of the system. The first mode is observed to be almost 20 times higher than the second mode. Therefore, insight into the modes is essential to visualize and define the optimum parameters of the system. The entire spectra of the DMD analysis are shown in Fig. 11(c). From the figure, it is revealed that the spectrum is symmetrical about $\eta_{i}=0$. The parabolic arc represents the temporal and spatial scales.^27^ It is observed that a few modes are presented in the positive quadrant. The modes with low energy and very high frequency are insignificant for the stability of the fluid motion. In comparison, the eigenvalue presented under the inverted parabola is the least stable. Considering the Ritz value of λ=1, the real and imaginary eigenvalues are shown in Table IV. With the dominant first mode, the second and third modes contribute only 1/20 of the first mode.

**TABLE IV.** Eigenvalues and energies of the dominant dynamic mode.

Interaction	$\lambda_{r}$	$\lambda_{i}$	L_2_ norm
1	1	0	3.73 × 10^–6^
2	0.99	±0.02	1.86 × 10^–7^
3	0.98	±0.41	8.47 × 10^–7^

**FIG. 12.**
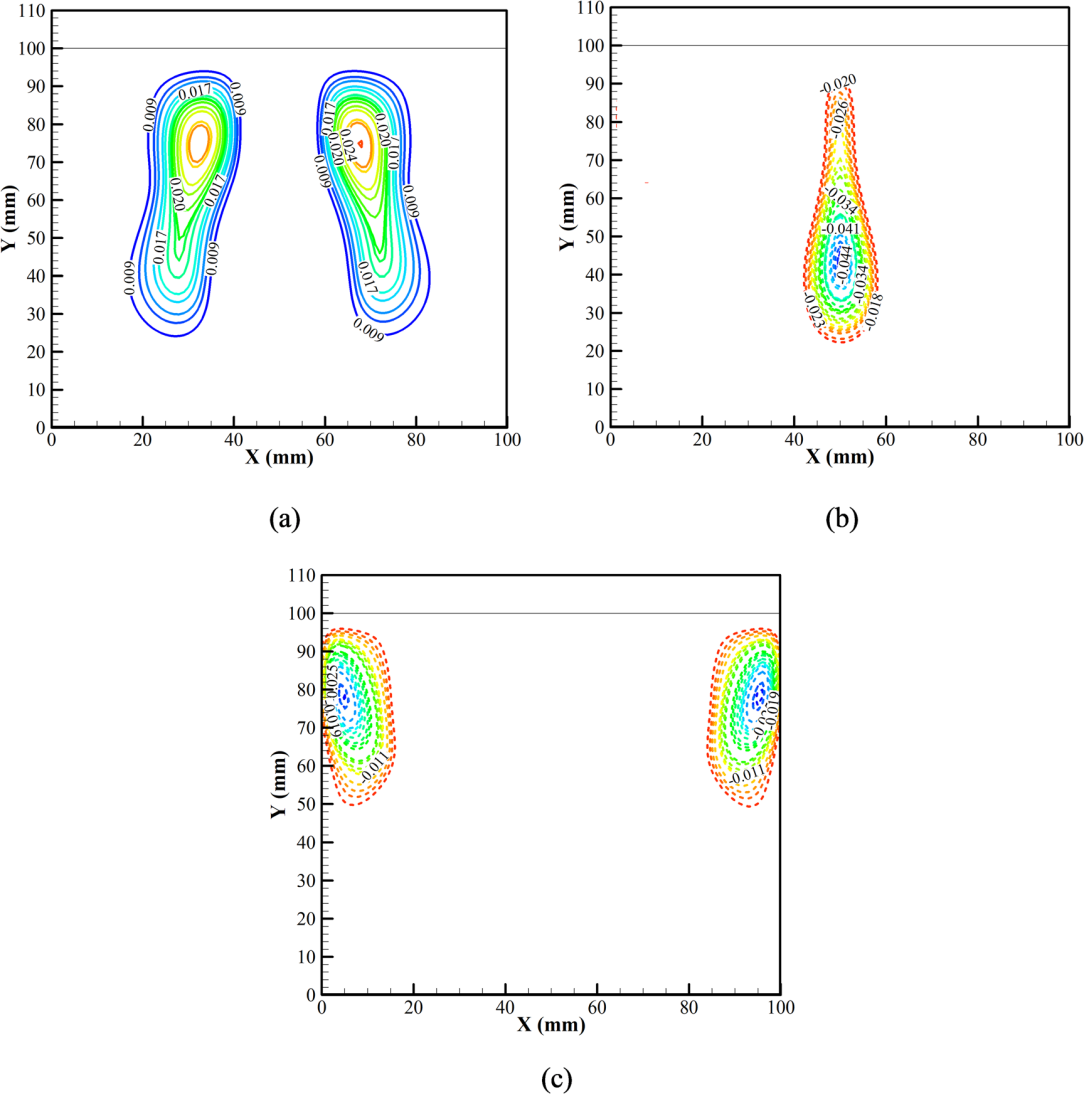
Dynamic modes of *v* velocity field of (a) the first mode, (b) the second mode, and (c) the third mode at the cooling phase.

The first three dominant modes for the *v* velocity field during the cooling phase are shown in Fig. 12. Figure 12(a) shows that the most dominant fluid interaction is between the Gd plate and the adjacent fluid, triggering the phenomenon of natural convection. The second most dominant fluid interaction is the secondary interaction of the fluid while participating in natural convection [Fig. 12(b)]. Thus, the first and the second dominant interactions significantly enhance the natural convection cycle inside the air chamber and thus promote cooling of the chamber. The third dominant fluid interaction can be seen due to the heat leak into the system [Fig. 12(c)]. Heat is allowed to leak into the system through the sidewall. The temperature rises, thereby inducing vortices into the flow, and heat is dissipated into the system. However, from the strength of all the induced vortices, the vortices due to the cool Gd plate and the adjacent air are dominant. Thus, cooling of the chamber is achieved.

**FIG. 13.**
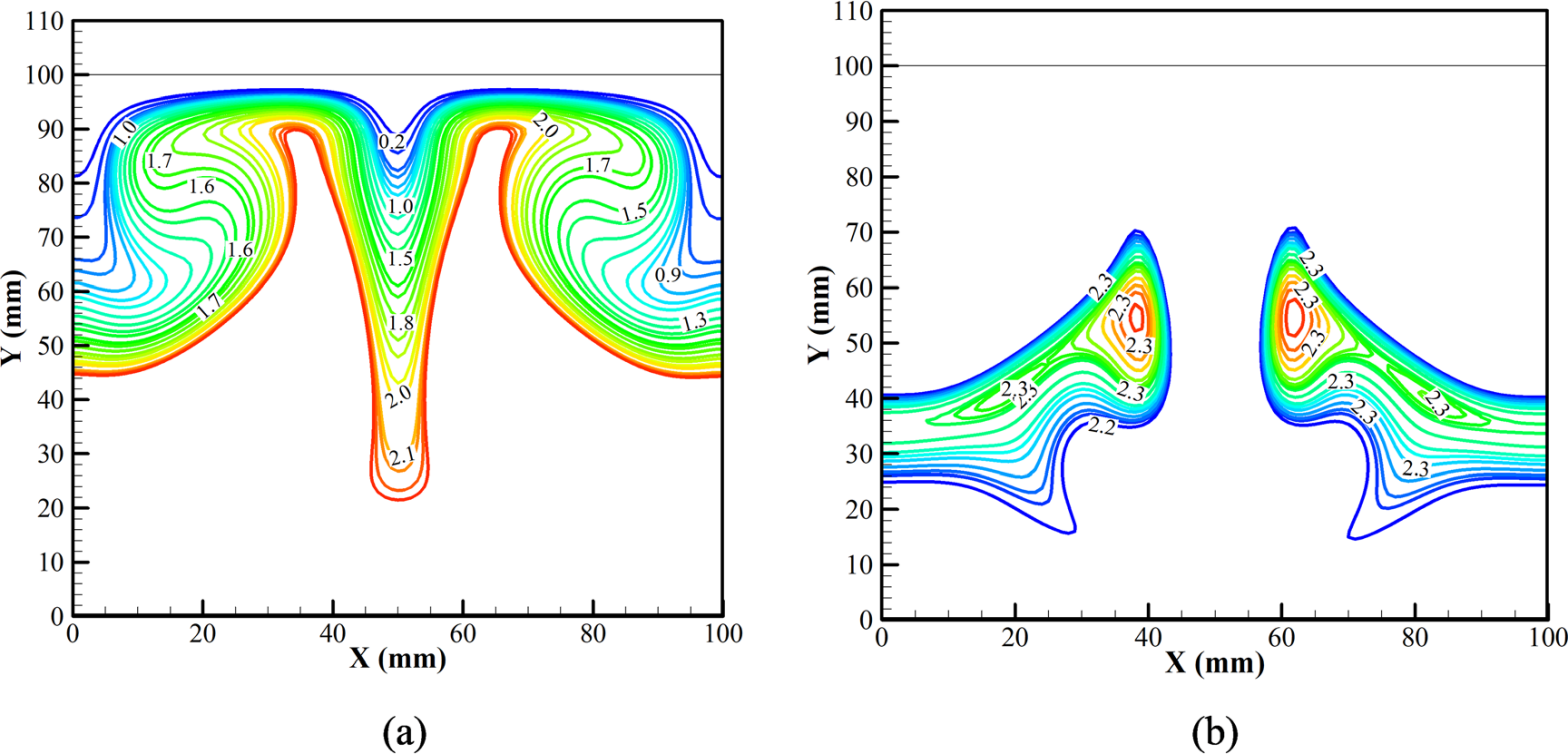
Dynamic modes of the isotherms field: (a) first mode and (b) second mode and at the cooling phase.

For better understanding, the DMD analysis is also carried out for a set of isotherm data during the same cooling phase, as shown in Fig. 13. The first dominant mode of the isotherms complements the first dominant mode of the *v* velocity field during the cooling phase Fig. 13(a). From Fig. 1(a), it is observed that relatively hot air rises, and the cool air descends, thereby setting natural convection in the enclosure. This set of interactions results in a higher slope of the temperature gradient. Followed by the first mode, the second mode is also presented in Fig. 13(b). Due to the secondary interaction, the isotherms are initiated due to natural convection triggered by the cooled Gd plate. Similar to the first mode of isotherms, the second mode also complements the second mode of the *v* velocity field during the cooling phase.

### A. Performance calculation

The performance of the vaccine storage system is evaluated to compare the present design with the other existing technologies. Using Eqs. (13) and (14), the cooling power using 0.08 kg of Gd and heat rejected during magnetization are evaluated. Therefore, the *COP* of the present system is calculated as 1.3 using Eq. (16). It can be noted from Eq. (16) that the *COP* can be improved by reducing the heat leakage to the vaccine chamber. If the magnetocaloric effect for the above system is switched off, the rise in temperature due to $Q_{l}=0.828\;W$ with the ice block of 0.75 kg is 0.0005 °C/s. Therefore, the life span of the current ice pack will be 1.03 h. For the current system, the ice pack is exposed to heating and cooling by Gd in a cycle of 18.2 s. However, with no load, as shown in Fig. 6, the temperature of the Gd sample falls below the initial temperature to a value of around −0.6 °C. Thus, the ice pack of 0.75 kg is self-sustained for a longer time with no load.

## CONCLUSION

The performance of the ice-based vaccine chamber is improved using the magnetocaloric effect. Magnetization and de-magnetization are performed over gadolinium blades of 0.08 kg. The magnetization process (0.3–1.3 T) for a time period of 0.1 s releases heat. The ice pack of 0.75 kg readily absorbs a part of this heat through an extended fin (aluminum) by conduction heat transfer. During the de-magnetization process for a time period of 0.1 s, the temperature of the Gd sample falls abruptly and produces a cooling effect by natural convection inside the vaccine chamber after the enclosure is left untouched for 15 s. From the DMD analysis, it is found that the most dominant fluid interaction occurs between the cooled Gd plate and the adjacent fluid. The strength of the interaction is approximately 20 times stronger than the second most dominant interaction, which is initiated due to the secondary interaction of the same phenomenon. The obtained cooling power using 0.08 kg of Gd is found as 31.514 W. The evaluated *COP* of the system is 1.3. Moreover, the improved traditional vaccine storage system with combined ice and magnetocaloric effect is cost-effective. Thus, using the integrated design of an ice pack and magnetic refrigeration, the ice pack life can be enhanced by 2.55 times. Thus, using the MCE in conjunction with a traditional ice-based system can be beneficial to roll out large vaccination drives. Moreover, the heat leak into the chamber is neutralized by the MCE, producing the necessary cooling effect. The reported configuration presents a thermal management system, which is self-reliant with no load condition. This auxiliary MCE cooling not only increases the performance of the system but also reduces human effort and ice consumption, which makes this system more suitable for challenging areas.

## Data Availability

The data that support the findings of this study are available from the corresponding author upon reasonable request.

## NOMENCLATURE

$A_{w}$: Area of the sidewall (m^2^)
$c_{a}$: Specific heat of air (J kg^−1^ K^−1^)
$c_{i}$: Specific heat of ice (J kg^−1^ K^−1^)
*COP*: Coefficient of performance
$k_{w}$: Conductivity of the side insulator wall (W m^−1^ K^−1^)
$m_{a}$: Mass of air (kg)
$m_{i}$: Mass of ice (kg)
$Q_{c}$: Cooling power (W)
$Q_{e}$: Electric power (W)
$Q_{l}$: Heat leak into the system (W)
$Q_{r}$: Heat released to ice (W)
$S_{}$: Total entropy ( $J\;{\mathrm{mol}}^{-1}\;K^{-1}$)
$S_{\mathrm{ele}}$: Entropy (electronic) ( $J\;{\mathrm{mol}}^{-1}\;K^{-1}$)
$S_{\mathrm{lat}}$: Entropy (lattice) ( $J\;{\mathrm{mol}}^{-1}\;K^{-1}$)
$S_{\mathrm{mag}}$: Entropy (magnetic) ( $J\;{\mathrm{mol}}^{-1}\;K^{-1}$)
*T*: Temperature (°C)
$T_{c1}$: Cycle end temperature (K)
$T_{c2}$: Cycle start temperature (K)
$T_{i1}$: Ice final temperature (K)
$T_{i2}$: Ice initial temperature (K)
$T_{in}$: Inside temperature (°C)
$T_{\mathrm{out}}$: Outside ambient temperature (°C)

**Greek symbols**
ν: Kinematic viscosity of air (m^2^/s)
ρ: Density of air (kg/m^3^)

**Abbreviations**
Gd: Gadolinium
I1: Induced vortex due to the interaction of vortex W1 and wall
I2: Induced vortex due to the interaction of vortex W2 and wall
MCE: Magnetocaloric effect
P1: Left produce vortex, produce during natural convection
P2: Right produce vortex, produce during natural convection
W1: Produce vortex due to the left vertical wall
W2: Produce vortex due to the right vertical wall
